# Transport of Young Veal Calves: Effects of Pre-transport Diet, Transport Duration and Type of Vehicle on Health, Behavior, Use of Medicines, and Slaughter Characteristics

**DOI:** 10.3389/fvets.2020.576469

**Published:** 2020-12-18

**Authors:** Francesca Marcato, Henry van den Brand, Bas Kemp, Bas Engel, Maaike Wolthuis-Fillerup, Kees van Reenen

**Affiliations:** ^1^Adaptation Physiology Group, Wageningen University & Research, Wageningen, Netherlands; ^2^Wageningen Livestock Research, Wageningen University & Research, Wageningen, Netherlands; ^3^Biometris, Wageningen University & Research, Wageningen, Netherlands

**Keywords:** transport, health, behavior, slaughter characteristics, medicine use, veal calves

## Abstract

The aim of this study was to investigate effects of different early life transport-related factors on health, behavior, use of medicines and slaughter characteristics of veal calves. An experiment was conducted with a 2 × 2 × 2 factorial arrangement with 3 factors: (1) provision of rearing milk or electrolytes before transport, (2) transport duration (6 or 18 h), and (3) type of vehicle (open truck or conditioned truck). The study included male Holstein-Friesian and cross-bred calves (*N* = 368; 18 ± 4 days; 45.3 ± 3.3 kg). Data on health status of calves were collected at the collection center and at the veal farm until week 27 post-transport. Behavior of calves was recorded during transport and at the veal farm until week 13 post-transport. Use of herd and individual medical treatments was recorded at the veal farm. The prevalence of loose or liquid manure at the veal farm from day 1 until week 3 post-transport was lower in electrolyte-fed calves transported in the conditioned truck compared to electrolytes-fed calves transported in the open truck or milk-fed calves transported in both the conditioned and open truck (Δ = 11% on average; *P* = 0.02). In comparison with the open truck, calves transported in the conditioned truck had lower prevalence of navel inflammation in the first 3 weeks post-transport (Δ = 3 %; *P* = 0.05). More milk-fed calves received individual antibiotic treatments compared to electrolyte-fed calves at the veal farm (*P* = 0.05). In conclusion, the transport-related factors examined in the present study affected health and behavior of calves in the short-term, but there was no evidence for long-term effects. It remains unknown why no long-term effects were found in this study. Perhaps this absence of transport-related effects was due to multiple use of medical treatments in the first weeks at the veal farm. Alternatively, it might be that the collective effects of the transition from the dairy farm to the veal farm, and of the husbandry conditions during the subsequent rearing period, on the adaptive capacity of calves were so large that effects of individual transport-related factors were overruled.

## Introduction

Calves at Dutch veal farms are usually collected from different dairy farms, including dairy farms from other EU-countries (especially Germany) (1). Collection procedures, which involve mixing of calves from multiple sources, transport to a collection center and subsequent transport to the veal farm result in stress and disease challenges (2, 3). Additionally, placement of calves into a new housing facility and their adaptation to a new feeding regime might also contribute to health problems (3). Transport normally occurs in the first weeks of the life of calves (14–20 days of age) when they are highly susceptible to microorganisms against which they have no colostral antibodies (4, 5). Poor condition of calves directly post-transport (calves with failure of passive transfer of immunity, dehydration and navel inflammation) is negatively linked to long-term performance of calves (6, 7). A high dehydration score, sunken flanks, diarrhea and navel infection upon arrival at the veal farm are related to mortality in the first 21 days post-transport (8). Dehydration may result from feed and water withdrawal around transport and is associated with body weight losses. Severe body weight loss during transport (over 10%) increases the risk of lameness and mortality in calves (6, 9). Transport is also related to incidence of respiratory diseases in calves after arrival at a feedlot (10). Overall, transport is a challenge for young veal calves, but it remains unknown which specific transport-related factors play a dominant role. Several transport-related factors have an effect on health (8) and behavior (e.g., standing vs. lying), thus influencing the recovery time of young calves during and in the immediate post-transport period (11). In a previous study (12), we examined the effects of pre-transport diet (milk vs. electrolytes), transport duration (6 vs. 18 h), and type of vehicle (open truck vs. conditioned truck) on the physiological status of young veal calves at the beginning of the rearing period. The aim of the current study was to investigate the effects of these transport-related factors on health (including the use of medicines) during the entire rearing period, behavior and slaughter characteristics of calves at the veal farm. We hypothesized that feeding milk, transportation of calves for 6 h in a conditioned truck, and likely the interaction between these factors, might contribute to less health problems and behavioral signs of discomfort compared to the other treatments.

## Materials and Methods

### Experimental Overview

The experiment had a 2 × 2 × 2 factorial arrangement, including the following factors: (1) provision of rearing milk or electrolytes prior to transport; (2) transport duration (6 or 18 h); (3) type of vehicle (open truck or conditioned truck). The experiment included 368 bull Holstein Friesian and crossbred calves [18 ± 4 days; 45.3 ± 3.3 kg body weight (BW)], transported over two consecutive weeks (*N* = 184 calves/week). Calves were transported from a collection center in Bocholt-Barlo, Germany, where they stayed from 4.00 a.m. until 14.00 p.m., to a Dutch veal farm in Veghel. All animals followed current practices, including handling and mixing procedures at the collection center and transportation, and all calves were in compliance with the minimal weight and health requirements [BW > 36 kg; age: minimum 14 days; no signs of disease and injury (13)]. The experiment was approved by the Central Committee on Animal Experiments (the Hague, the Netherlands; Approval Number 2017.D-0029).

### Handling of Calves at the Collection Center, During Transport and at the Veal Farm

At the collection center, calves were randomly allocated to one of the eight treatment groups by the manager. Calves were fed *via* a bucket with nipples, with 1.5 l of rearing milk (125 g of milk powder/l; per kg of milk powder: ME = 4028 kcal, CP = 190 g, crude fat = 157 g, digestible lysine = 18.7 g; made with plant-based ingredients; Tentofok KO, Tentego, The Netherlands) or a mixture of electrolytes (20 g of electrolytes/l of water; per 100 g of powder: Na = 7.3 g and moisture = 3.8 g; Navobi, Staverden, The Netherlands) dissolved in 1.5 l water.

After feeding, calves rested for ~2 h and thereafter they were loaded on the vehicle. The vehicle consisted of two parts: the truck was conditioned, which means it was provided with a side-ventilation system, it was isolated, and the climate was controlled regarding in and outlet of air (KVM Livestock Transport System^TM^, Kleventa BV, Lichtenvoorde, The Netherlands). Settings were according to those provided by the manufacturer and applied by the transporter. The trailer was regular, open and lacked a ventilation system or climate control. Temperature and relative humidity in both vehicles are shown in Appendix 1. Both truck and trailer were divided into four compartments with straw bedding, two at the lower deck (3.60 m length × 2.45 m width × 1.35 m height) and two at the upper deck (3.60 m length × 2.45 m width × 1.45 m height). Each compartment contained 23 calves of one treatment group at the same stocking density (0.383 m^2^ per calf). Treatments were distributed in the vehicle according to a design that allows for estimation of all main effects and relevant interactions [for details see Marcato et al. (12)]. After loading, transport was conducted by two drivers, switching every 3 h. Neither food nor water was provided to calves during transport. After 6 h transport, the truck arrived at the veal farm and all calves were unloaded. Calves assigned to 6 h transport were placed in the veal farm, whereas the calves assigned to 18 h transport were reloaded on the truck and trailer (in the same compartments as before) and transported for another 12 h. Calves in the 6 and 18 h transport treatment groups were appropriately distributed across the truck and trailer and, therefore, located in both upper and lower decks [see Marcato et al. (12)]. Unloading calves located in an upper deck after 6 h of transport required that calves located in the lower deck had to be unloaded first; in some instances these latter animals belonged to the 18 h transport treatment group. In order to avoid unwanted confounding between transport duration and unloading and reloading of calves in part of the calves subjected to 18 h of transport, we decided to unload all calves after 6 h of transport and subsequently reload the animals in the 18 h transport treatment group, placing them in the appropriate compartment. At the veal farm, calves were distributed across 64 pens that were divided over 8 similar compartments. Each compartment included 8 pens, with 5 or 6 calves per pen. Treatments were randomly distributed across pens in every compartment. Calves were housed individually within each pen for the first 3 weeks post-transport. Subsequently, calves were kept in groups.

### Calculation of the Sample Size

The number of experimental units required in the present study was based on a power analysis. Our experimental design was based on the principle that pen (or group) was the basic, independent experimental unit. We have extensive experience with multifactorial experiments with veal calves, and in one of the previous studies we used 16 pens per level of main effects, and 4 pens per treatment combination (14). Using this setup, we were able to detect differences between two treatment levels of about one unit standard deviation (SD) with a power of 0.80. However this latter experiment was performed on an experimental farm, under relatively standardized conditions, and using a specific and relatively standardized subset of calves. We anticipated that both the variation in conditions and between calves would be higher during the current experiment which took place under commercial conditions. A recent power analysis that we performed using a very large data set with carcass weights recorded both under experimental and commercial conditions (15) supported this latter assumption, and suggested that under commercial conditions the SD could be 1.5 times higher than under more controlled experimental conditions. Power analysis showed that in order to maintain the same statistical power, the number of experimental units should be approximately doubled. Therefore, in the present experiment, we used 32 pens per level of main effects, and 8 pens per treatment combination.

### Health Assessment

Health assessment of calves was performed at the collection center (during the resting period), and at the veal farm, on day 1, and in weeks 1, 3, 5, 7, 9, 11, 13, 15, 17, 19, 21, 23, 25, and 27 post-transport by two observers. Inter-observer reliability was tested before the experiment for both health and behavioral observations (k-coefficient = 98%). Two protocols were used for health assessment. The first one, shown in Appendix 2, was used at the collection center and at the veal farm from day 1 until week 3, when the calves were housed individually. The second protocol was according to the one used by Brscic et al. (5) based on the Welfare Quality® Protocol on veal calves, and appropriate for use at pen level. This latter protocol was used to clinically score calves from week 5 until 27 post-transport (Appendix 3).

### Processing of Health Data

Appendices 2, 3 show a complete list of health variables assessed at the collection center and at the veal farm, throughout the entire rearing period. Each health variable was first expressed at pen level as a percentage reflecting the number of calves displaying a health problem divided by the number of calves in the pen (Tables 1, 2). These percentages were averaged per treatment. Prior to statistical analyses, some health variables collected until week 3 post-transport were grouped as follows: (1) navel inflammation = navel with score 1 and 2; (2) loose or liquid manure = loose manure (with score 1) and liquid manure (with score 2); this category includes either infectious diarrhea or feeding-related loose or liquid manure, but it was not possible to make this distinction based on the visual clinical assessment.

**Table 1:** Effects of pre-transport diet, type of vehicle and transport duration on health variables of young veal calves assessed at day 1 after arrival at the veal farm.

	**Pre-transport diet**				**Type of vehicle**				**Transport duration**			
**Parameter**	**Electrolytes**	**Milk**	**SE^**a**^**	***P*-value**	**Conditioned truck**	**Open truck**	**SE**	***P*-value**	**6 h**	**18 h**	**SE**	***P*-value**
Navel inflammation	8.9	9.7	2.2	0.21	6.7	11.9	2.1	0.16	9.9	8.6	2.1	0.05
Eye discharge	7.1	6.3	2.1	0.39	5.0	8.4	2.0	0.49	6.1	7.3	2.1	0.76
Sunken eyes	38.3	40.9	3.2	0.63	35.7	43.5	3.2	0.08	36.5	42.8	3.2	0.31
Drooped ears	9.6	12.9	2.0	0.08	11.1	11.3	2.0	0.83	9.7	12.8	2.0	0.69

*All parameters are expressed as an average proportion at pen level (N = 5 or 6 calves/pen). Data are shown as raw means ± SE^a^*.

a *SE, standard error*.

**Table 2:** Effects of pre-transport diet, type of vehicle and transport duration on health variables of young veal calves assessed from day 1 until week 3 after arrival at the veal farm.

	**Pre-transport diet**				**Type of vehicle**				**Transport duration**			
**Parameter**	**Electrolytes**	**Milk**	**SE^**a**^**	***P*-value**	**Conditioned truck**	**Open truck**	**SE**	***P*-value**	**6 h**	**18 h**	**SE**	***P*-value**
Navel inflammation	8.0	7.2	1.2	0.93	5.9	9.2	1.2	0.05	8.3	6.8	1.2	0.04
Joint problems	2.3	1.3	0.8	0.57	1.5	2.1	0.8	0.37	1.9	1.8	0.8	0.17
Loose or liquid manure	22.8	30.7	2.6	<0.01	24.8	28.7	2.6	0.30	26.1	27.4	2.6	0.15
Eye discharge	7.1	6.2	1.1	0.63	6.5	6.9	1.1	0.75	6.7	6.6	1.1	0.80
Sunken eyes	39.0	40.3	2.5	0.52	38.6	40.7	2.5	0.70	39.8	39.5	2.5	0.71
Drooped ears	13.9	13.3	1.6	0.80	13.4	13.8	1.6	0.92	13.7	13.5	1.6	0.39

*All parameters are expressed as an average proportion at pen level (N = 5 or 6 calves/pen). Data are shown as raw means ± SE^a^*.

a *SE, standard error*.

To qualitatively compare health data recorded at the collection center with those recorded at the veal farm, a proportion was calculated as follows: (sum of calves displaying a health problem/total number of calves) × 100 (see Table 3).

**Table 3:** Severity of health problems at the collection center, on day 1 post-transport and in the first 3 weeks at the veal farm.

	**Place and time**		
**Health variables**	**Collection center: before transport**	**Veal farm: day 1**	**Veal farm: day 1 until week 3**
Signs of pneumonia	2.7	0.3	2.3
Eye discharge	3.8	6.5	6.6
Nasal discharge	1.9	0.5	3.9
Loose or liquid manure	5.4	5.2	26.7
Navel inflammation	6.5	9.2	7.4
Sunken eyes	18.7	39.7	39.4
Joint problems	2.1	0.5	1.7
Drooped ears	5.4	11.1	13.5

*Values represent proportions at batch level calculated as following: (the number of calves displaying a health problem/the total number of calves) × 100*.

### Behavioral Observations

The first behavioral observations were conducted at the collection center during the resting hours after the application of the feeding treatment. Two observers conducted behavioral observations, using the scan sampling technique according to Martin et al. (16). Behavior of calves was assessed every 5 min for 1 h according to an adapted version of the ethogram used by Webb et al. (17) (Appendix 4). After the rest period, calves were loaded in the truck and trailer according to their respective treatments. Every compartment of both truck and trailer contained a camera that recorded standing and lying behavior throughout the 6 and 18 h of transport. Behavior was also assessed at the veal farm where cameras (*N* = 8, each positioned in every compartment of the stable) recorded standing vs. lying behavior during the first 24 h after arrival. In addition, two observers assessed behavior of calves by direct observations and using an instantaneous scan sampling technique at 5 min intervals for 1 h. These direct observations were done after arrival of calves, and in weeks 1, 3, 5, 9, and 13 post-transport (always after feeding). Behavioral variables shown in Appendix 4 were grouped into 3 main categories prior to statistical analyses: (1) comfort behavior = licking another calf, self-grooming, rubbing, chewing, eating, and drinking; (2) discomfort behavior = tongue playing, manipulating objects, manipulating another calf, urine drinking, and repetitive calling; (3) playing behavior = mount/leap/jump/back/turn, head-butt, running.

### Use of Medicines

Use of antibiotics and other medicines during the entire rearing period was recorded at the level of both herd and individual calf. Information on individual treatments included the following data: (1) whether the calf was treated or not with antibiotics or other medicines (this category included products such as anti-inflammatories, multivitamins, anti-coccidiosis, with the exclusion of antibiotics) during the rearing period; (2) single or repeated antibiotic/medical treatments during the rearing period; (3) age at which treatments were applied; (4) type of antibiotic or medication used. Herd treatments (applied on all calves, *via* the milk) were also recorded, including the age at which they were applied and the type of medication used.

### Slaughter Characteristics

Slaughter characteristics were assessed per calf and included carcass weight (kg), color of the meat (scale 1–10 points, from pale to dark red color), fat coverage (scale 1–5 points, from low to very high fat coverage) and conformation class (scale 1–15 points, from excellent to poor carcass quality) (18).

### Statistical Analyses

All data were analyzed in SAS 9.4 (SAS Inst. Inc., Cary, NC). Health and behavioral data (expressed as a proportion of health problems or behaviors per pen) determined on day 1 and directly post-transport, respectively, were analyzed with a generalized linear mixed model with Pseudo Likelihood or equivalently Penalized Quasi Likelihood (PQL) (19), employing SAS procedure GLIMMIX. At this stage, calves were individually housed inside pens. The systematic part of the model comprised the following fixed effects:

μ + Batch_i_ + Uplo_j_ + Bafr_k_ + Diet_l_ + Type_m_ + Duration_n_ + (Diet_l_ × Duration_n_) + (Diet_l_ × Type_m_) + (Duration_n_ × Type_m_) + (Diet_l_ × Type_m_ × Duration_n_) (1)

Here, μ is a base level and Batch_i_ = batch (*i* = 1, 2), Uplo_j_ = position in the vehicle (j = upper or lower deck), Bafr_k_ = position in the vehicle (k = front or back), Diet_l_ = pre-transport diet (l = rearing milk or electrolytes), Type_m_ = type of vehicle (m = open or conditioned truck), and Duration_n_ = transport duration (*n* = 6 or 18 h) are main effects. The model also comprised two- and three-way interactions between diet, type of vehicle and transport duration. Interactions were considered not significant when *P* > 0.05. In addition, random effects for pen and compartment at the veal farm were included in the linear predictor. The logit link function was used in concert with the variance function of the binomial distribution, which included a multiplicative dispersion factor that was estimated from the data. Here and in subsequent analyses, for all fixed effects, approximate *F*-tests were used (20). Interactions that were not significant were excluded from the model (when higher order interactions were already excluded, i.e., respecting the hierarchy of interaction terms). Subsequent pairwise comparisons were done with Fisher's LSD method.

Health data and direct behavioral observations (expressed as proportion of health problems or behaviors per pen) assessed from the arrival of calves at the veal farm until week 3 post-transport were analyzed with a generalized linear mixed model (again PQL and GLIMMIX). Until week 3 post-transport, calves were still individually housed inside pens. The systematic part of the model comprised the following fixed effects:

μ + Batch_i_ + Uplo_j_ + Bafr_k_ + Diet_l_ + Type_m_ + Duration_n_ + Time_o_ + (Diet_l_ × Duration_n_) + (Diet_l_ × Type_m_) + (Duration_n_ × Type_m_) + (Diet_l_ × Time_o_) + (Duration_n_ × Time_o_) + (Type_m_ × Time_o_) + (Diet_l_ × Type_m_ × Duration_n_) (2)

in the same notation as before and additionally with Time_o_ = sampling moment (o = T0 for behavior or day 1 for health, week 1 and 3) as main effect. Three-way interactions between diet, type of vehicle and transport duration, and two-way interactions between pre-transport diet, type of vehicle transport duration and time were also included in the model. Interactions were considered not significant when *P* > 0.05. The model comprised random compartment effects. For the repeated measurements on the same pen a first order auto regressive model (based on the actual distance between time points) was adopted.

Health data assessed from week 5 until 27 and direct behavioral observations assessed from week 5 until 13 were also analyzed with the generalized linear mixed model (Equation 2). During this period, calves were housed in groups instead of individually. Between week 5 and 27 post-transport, the presence of loose or liquid manure, as well as thick and white manure, were recorded as a binary response at pen level (i.e., present or not present). These variables were also analyzed with the generalized linear mixed model (Equation 2).

Data on individual treatments with antibiotics and other medicines during the entire rearing period were expressed as binary data (0 = calf not treated at individual level with antibiotics or medicines; 1 = calf treated at least once at individual level with antibiotics or medicines during the rearing period). These data were analyzed with a generalized linear mixed model (analysis with PQL and GLIMMIX) similar to model 1, but for binary data.

Continuous data on carcass weight at slaughter were analyzed with a linear mixed model (analysis with restricted maximum likelihood with SAS procedure PROC MIXED) with fixed and random effects as in Equation 1 and additional normally distributed error (or residual) terms. Residuals were checked for normality and homogeneity of variance and data were log transformed when deemed necessary.

Carcass weight was also analyzed in relation to the number of individual medical treatments. The number of individual medical treatments was introduced as a qualitative factor in the model 1, comprising three main levels: 0 = calf not treated; 1 = calf treated once or twice; 2 = calf treated > 2 times.

In all analyses, effects with *P* ≤ 0.05 were considered significant, whereas those with 0.05 < *P* < 0.10 were considered as a tendency toward significance.

## Results

The results of the present study will be shown in four main domains: health, behavior, use of medicines and slaughter characteristics. In each of these domains, effects of main factors, which included pre-transport diet, transport duration and type of vehicle, will be reported. Three-way and two-way interactions were never significant, with the exception of the interaction between pre-transport diet and type of vehicle on loose and liquid manure which is described in the first paragraph.

### Health

The day post-transport, there were no significant effects of treatments on individual health parameters (Table 1). Drooped ears tended to be higher in milk-fed calves than in electrolytes-fed calves (Δ = 3.3%; *P* = 0.08) and sunken eyes tended to be higher in calves transported in the conditioned truck than in calves transported in the open truck (Δ = 7.8%; *P* = 0.08).

For the average prevalence of loose or liquid manure from day 1 until week 3 post-transport there was an interaction between pre-transport diet and type of vehicle. The percentage of calves with loose or liquid manure was lower (18%) in electrolytes-fed calves transported in the conditioned truck compared to electrolytes-fed calves transported in the open truck (28%) and milk-fed calves transported in both the conditioned and open truck (31% on average; *P* = 0.02). The percentage of calves with navel inflammation was higher in calves transported in the open truck and calves transported for 6 h compared to calves transported in the conditioned truck and calves transported for 18 h (Δ = 3.3% and Δ = 1.5%, respectively; *P* ≤ 0.05; Table 2).

Prevalences of navel inflammation and loose or liquid manure changed significantly in the first 3 weeks post-transport (*P* < 0.01). Navel inflammation decreased from day 1 (9%) until week 3 (4%), whereas loose or liquid manure gradually increased in this period (from 5% on day 1 to 39% in week 3). In addition to the effects of time on health problems in the first 3 weeks post-transport, Table 3 shows the trend of health problems from the collection center until week 3 post-transport. Overall, the prevalence of the majority of health problems gradually increased in the period between the collection center and week 3 post-transport. Prevalences of loose or liquid manure and sunken eyes in the first 3 weeks post-transport more than doubled compared to the same prevalences at the collection center (Δ = 22% and Δ = 20%, respectively).

**Figure 1:**
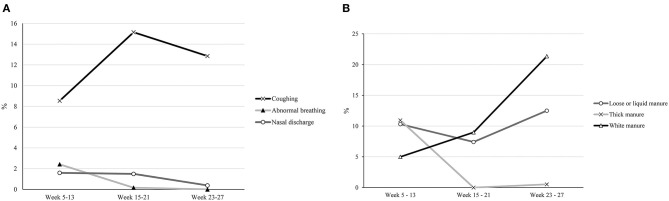
**(A)** Prevalence of coughing, abnormal breathing and nasal discharge in veal calves between week 5 and 27 post-transport (expressed at average proportions at pen level). **(B)** Prevalence of loose or liquid manure, thick manure and white manure in veal calves between week 5 and 27 post-transport (expressed as average proportions of pens).

Overall, prevalences of health problems from week 5 until 27 were relatively low (<10%), with the exception of coughing (12%), and there were no significant differences due to transport factors. As shown in Figure 1A, the prevalence of coughing changed significantly in this period (*P* < 0.01) and was highest between week 15 and 21 post-transport (15%). Besides coughing, abnormal breathing and nasal discharge, the other two clinical signs of respiratory disease, were below 5%. Raw means for all three signs of respiratory disease beyond week 5 at the veal farm are also shown in Table 4. Figure 1B shows the prevalence of gastrointestinal problems at the veal farm. Thick manure was present only from week 5 until 13 (average prevalence 11%), whereas it disappeared in the remaining part of the fattening period. The average prevalence of loose or liquid manure from week 5 until 13 was 10%, decreased slightly (Δ = −3%) from week 15 until 21, and increased again (Δ = 5%) from week 23 until 27. White manure substantially increased during the entire rearing period (from 5 to 21%).

**Table 4:** Raw means recorded for coughing, abnormal breathing and nasal discharge in veal calves between week 5 and 27 post-transport.

	**Weeks post-transport**											
**Health variable**	**Week 5**	**Week 7**	**Week 9**	**Week 11**	**Week 13**	**Week 15**	**Week 17**	**Week 19**	**Week 21**	**Week 23**	**Week 25**	**Week 27**
Coughing	6.1 ± 1.2^a^	5.3 ± 1.2	9.8 ± 1.5	7.7 ± 1.3	13.7 ± 1.5	16.3 ± 1.6	15.1 ± 2.1	15.8 ± 1.8	13.4 ± 1.6	19.1 ± 1.9	10.1 ± 1.7	9.3 ± 1.5
Abnormal breathing	2.6 ± 0.8	2.7 ± 1.0	5.4 ± 1.1	0.3 ± 0.3	1.1 ± 0.6	0.4 ± 0.4	0.3 ± 0.0	0	0	0	0	0
Nasal discharge	2.6 ± 1.0	0.8 ± 0.6	1.3 ± 0.6	0.5 ± 0.4	2.7 ± 0.8	4.6 ± 1.3	1.1 ± 0.7	0	0.3 ± 0.3	1.1 ± 0.6	0	0

*All health variables are expressed as an average proportion at pen level (raw means ± SE^a^)*.

a *SE, standard error*.

### Behavior

During transport (61 vs. 39%) and directly post-transport (77 vs. 23%), calves spent most of the time lying compared to standing, but no significant differences were found between treatment groups. On the day post-transport, calves transported for 18 h showed more signs of discomfort compared to calves transported for 6 h (9 vs. 6%; *P* < 0.01). Additionally, calves transported in the conditioned truck showed more signs of discomfort behavior compared to calves transported in the open truck (9 vs. 5%; *P* = 0.01).

During the first 3 weeks post-transport, calves increased their time in a standing position (from 23% on day 1 to 51% in week 3 post-transport), and calves showed a gradual increase in comfort behavior (from 5% on day 1 to 15% in week 3 post-transport) and a decrease in discomfort behavior within this time frame (from 7% on day 1 to 4% in week 3 post-transport) (*P* < 0.01).

In the period between week 5 and 13 post-transport, comfort behavior gradually increased (from 30 to 53%) (*P* < 0.01). Play behavior increased up to a 4% in week 9 and subsequently, it decreased to 1% in week 13 post-transport (*P* < 0.01).

### Use of Medicines

The percentage of calves individually treated with antibiotics at least once during the rearing period at the veal farm was 33%. Among this fraction of calves, 70% of animals were treated once, 21% were treated twice and 9% were treated more than twice during the rearing period. More milk-fed calves received individual antibiotic treatments compared to electrolyte-fed calves throughout the rearing period (38 vs. 28%, respectively; *P* = 0.05). The percentage of calves that received at least one other medical treatment during the rearing period was 18%. Among this fraction of calves, 69% of animals were treated once, 23% were treated twice and 8% were treated more than twice. No significant differences were found between treatment groups on the use of other medical treatments. In the first 6 weeks at the veal farm, 25% of calves were individually treated with antibiotics and 22% of calves were treated with other medicines. In the following 6 weeks, calves were still individually treated for antibiotics (23%) and for other medicines (4%), but from week 13 until 27 calves were not treated at all, neither individually nor batch-wise. Besides individual treatments, calves were subjected to 5 herd treatments (on day 3, 13, 22, 37, and 47) with oxytetracycline HCl (1.43 g/100 kg/twice a day), doxycycline (1 g/100 kg/day), Tilmovet 250 mg/ml (5.45 ml/100 kg/twice a day), Ampisol 100% (2.26 g/100 kg/day), and doxycycline (0.58 g/100 kg), respectively. These herd treatments were provided *via* the milk for an average of 11 feedings per herd treatment.

### Slaughter Characteristics

**Figure 2:**
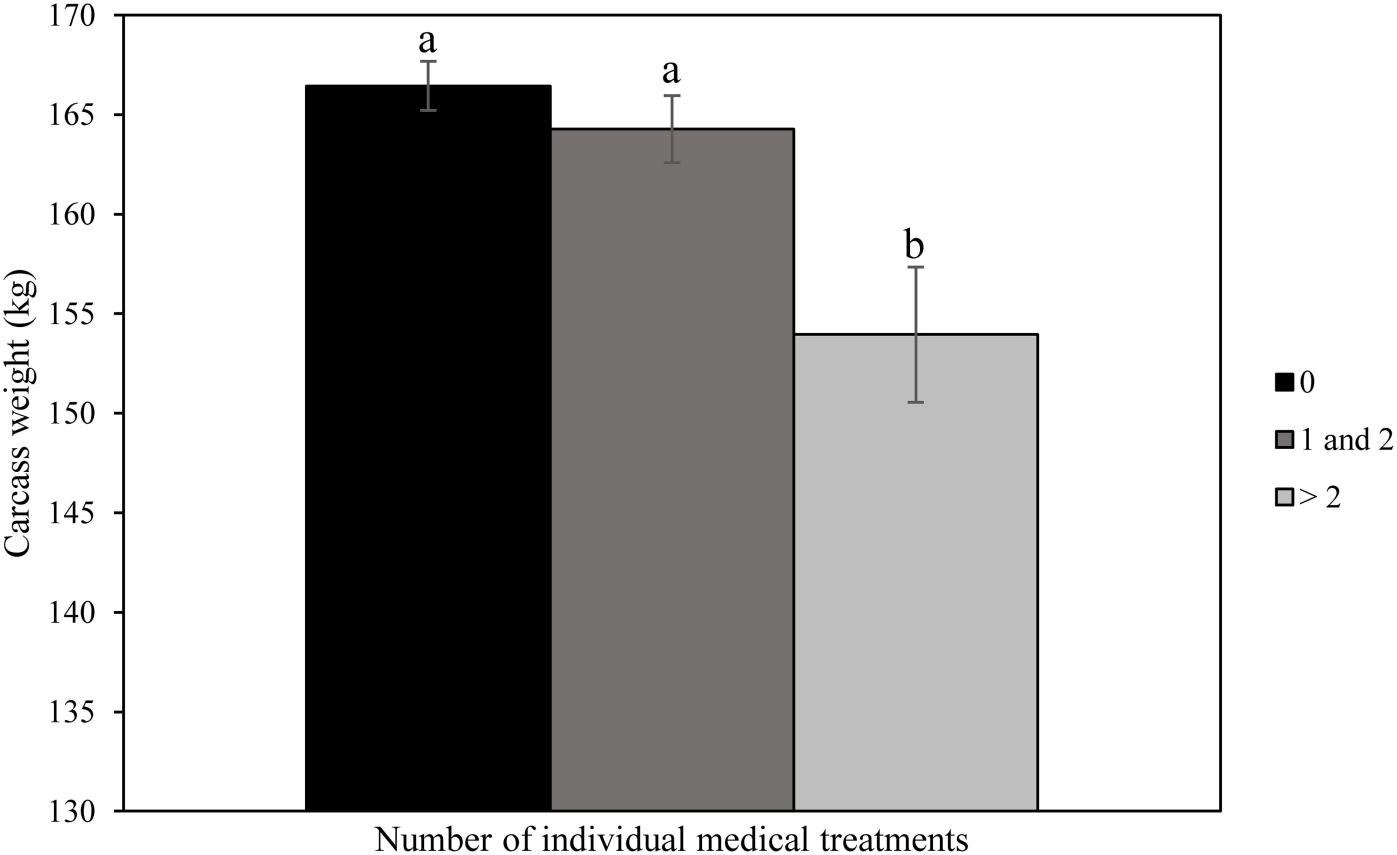
Effects of number of individual medical treatments of veal calves throughout the rearing period on carcass weight at slaughter.

**Figure 3:**
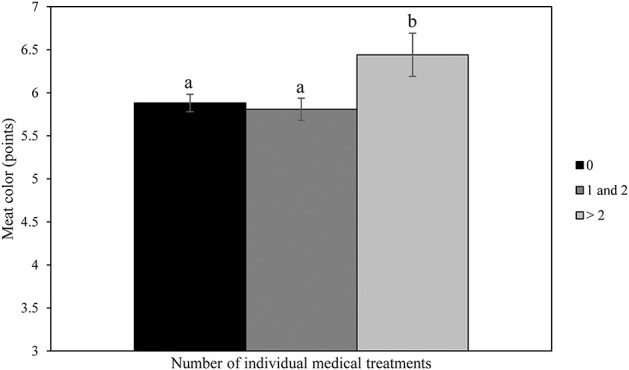
Effects of number of individual medical treatments of veal calves throughout the rearing period on meat color at slaughter.

No significant differences were found between treatment groups in relation to carcass weight (164.7 kg ± 18.4; range: 96–215 kg), conformation class (11.9 points ± 1.0; range: 8–15) and color of the meat (5.9 points ± 1.3; range: 2–10) at slaughter. Figure 2 shows a significantly lower carcass weight of calves receiving > 2 individual medical treatments compared to carcass weight of calves not treated or treated once or twice (*P* < 0.01). Figure 3 shows That the color of the meat of calves receiving > 2 individual medical treatments tended to be darker than the meat of calves not treated or treated once or twice (*P* = 0.06).

## Discussion

### Health and Use of Medicines

In the current study, health problems in young veal calves increased in the post-transport period compared to pre-transport values at the collection center. Thus, transport (including mixing and handling procedures and feed withdrawal) and the adaptation of calves to the new housing and feeding system at the veal farm were challenges for calves. Health outcomes measured at the veal farm were expressed at pen level, although calves were individually housed in the first 3 weeks post-transport. We used this approach because individually housed calves were not randomly distributed across the barn, but housed pen-wise, thus each pen contained 5 or 6 calves in adjacent baby boxes. Most of the effects of transport-related factors were evident in the first 3 weeks after arrival at the veal farm; this is also the period in which most of the medical treatments were applied. The day post-transport, the prevalence of sunken eyes, which is a clinical characteristic related to dehydration, was lower than the prevalence rate shown by Wilson et al. (21) (40 vs. 61%, respectively). Dehydration is associated with different factors, including transport and diarrhea (22, 23). In a previous study (12) we reported that up to 70% of the calves used in the current experiment were dehydrated (based on skin elasticity) already before transport. This explains why application of the treatments resulted in a large number of calves with sunken eyes upon arrival.

Pre-transport diet fed at the collection center had an impact on loose or liquid manure in the first 3 weeks post-transport, where milk-fed calves showed more loose or liquid manure than electrolyte-fed calves. Feeding milk prior to transport is a good remedy against energy depletion or hypoglycemia (24), which was also visible on most of energy related parameters measured in blood of calves in this experiment (12). However, time feeding milk may also contribute to alterations in fecal consistency related to transport stress and consequently intestinal atrophy, whereas feeding electrolytes is a good approach to treat calves displaying metabolic acidosis and diarrhea (24, 25). The different composition of the pre-transport diet might also explain the higher use of antibiotics in milk-fed calves compared to electrolyte-fed calves in the present experiment. Feeding more nutrients, especially before a challenge, such as transport, may increase fecal abnormalities in pre-weaned calves in the first weeks of life (26) and it may result in higher antibiotic use (27). However, since we were not able to make the distinction between infectious or feed-related diarrhea during our clinical assessment, we cannot rule out the possibility that some of the antibiotic treatments in the current study were applied without a proper clinical justification. This may have affected the difference in antibiotic treatments between milk-fed and electrolyte-fed calves. The significant interaction between pre-transport diet and type of vehicle means that the prevalence of loose or liquid manure was lowest in electrolyte-fed calves transported in the conditioned truck in comparison with electrolyte-fed calves transported in the open truck, and milk-fed calves transported in both the conditioned and open truck. Apparently, in combination with the pre-transport diet, the environment in the conditioned truck exerted some kind of protective effects on the likelihood of calves exhibiting loose or liquid manure during the first 3 weeks of the rearing period. As indicated above, however, we do not know whether this decrease concerns loose or liquid manure with an infectious or non-infectious origin.

In comparison with the open truck, transporting calves in the conditioned truck also reduced the prevalence of navel inflammation in the first weeks post-transport. At present, it remains unknown which environmental factors comprising the conditioned transport in our experiment (such as draft or differences between in and outlet airflow) contributed to these effects on calf health at the beginning of the rearing period; these environmental and climatic factors need to be defined and recorded in more detail in future research. In a recent study by Renaud et al. (8), involving close to 5,000 calves, navel inflammation at arrival was associated with early mortality at the veal farm (≤ 21 days post-transport). Therefore, the significant lower navel inflammation in calves transported in the conditioned truck found in the current experiment might be relevant at population level for reducing mortality at the veal farm, provided that conditioned transport would be used at a large scale.

In the first 3 weeks post-transport, navel inflammation decreased significantly and this is in line with Wilson et al. (21), although the starting prevalence rate in our study was lower (9 vs. 32%, respectively at the first sampling moment). Navel inflammation can be caused by environmental conditions during transport (e.g., lack of bedding on the truck, overcrowding) or by farm management practices before transport to the collection center (e.g., poor hygiene, lack of navel antisepsis) (6, 28). In the current experiment, it is likely that navel inflammation was already present at the dairy farm, because all calves were transported on a straw bedding and transport density was not high. Therefore, preventive measures at the dairy farms (high hygiene status, early intake of high-quality colostrum, navel dipping) are necessary to avoid this condition in the veal farm (6). Loose or liquid manure increased over time in the first 3 weeks at the veal farm. The prevalence of loose or liquid manure at day 1 post-transport was lower than reported by Wilson et al. (21) (5 vs. 16%), but higher in week 3 post-transport (39 vs. 17%). It appeared that, besides transport, calves struggled to adapt to a new feeding regime (based on milk replacer diets) at the veal farm. In addition to gastrointestinal problems, clinical signs of respiratory disease, gradually increased at the veal farm. Respiratory disease in white veal calves is often of a slow progressive nature, and likely due to presence of maternal immunity and frequently applied metaphylactic antimicrobial therapy (29). The starting prevalence of respiratory disease indicators was in line with the prevalence of bovine respiratory disease (BRD) shown by Pardon et al. (30) in the first 17 days post-transport at the veal farm (<5%). However, in Pardon et al. (30), 40% of calves showed signs of BRD at day 18 post-transport, a prevalence much higher compared to the 4% in the current experiment. However, prevalences obtained in different studies may be difficult to compare, because of differences in health protocols: the current experiment separately considered nasal discharge, coughing or abnormal breathing as clinical signs of respiratory disease, whereas Pardon et al. (30) defined BRD cases based on the simultaneous presence of depression, cough, higher rectal temperature and nasal discharge. Pardon et al. (31) reported peak prevalences of respiratory disease in veal calves between 2 and 6 weeks post-transport. In the current study, the highest prevalence of coughing occurred at a later stage (between week 15 and 21 post-transport) and this might be due to a reinfection of calves with respiratory pathogens after the first weeks post-transport (29). Next to respiratory disease, the prevalence of white manure and loose or liquid manure in the last weeks of the rearing period indicates that calves might struggle to adapt to the feeding scheme at the veal farm. Besides these health problems, there were no significant differences between the experimental treatments in prevalence of other health problems from week 5 until 27.

The current findings showed that health of calves destined to veal production can be already compromised at the collection center (as indicated by high prevalence of dehydration, sunken eyes and navel inflammation). Thus, in addition to transport-related factors such as examined in the present experiment, further attention on factors and (e.g., early rearing) conditions experienced by veal calves prior to arrival at a collection center is merited. The existence of relatively mild effects of transport factors on health problems in the immediate post-transport period, and the absence of significant effects in the longer term might be due to several reasons. First, it could be suggested that the transport and arrival at the veal farm as applied in the current experiment did not represent a severe enough challenge to significantly disturb the homeostasis of calves. However, this is highly unlikely given the profound overall effects of the experimental treatments on, for example, the physiological status (12) and a number of aspects of the clinical health of our calves (Table 3). Secondly, the collective effects of the transition of calves from the dairy to the veal farm (including transport and mixing with other calves at the collection center), and of the husbandry conditions during the subsequent rearing period (including dietary changes, and, again, mixing with other calves) might be so large that they overrule potential effects of individual transport factors as examined in the present experiment on health and adaptive capacity of calves. Thirdly, the high use of antimicrobials and medical treatments both at herd and individual calf level in the first 6 weeks of the rearing period may have masked potential effects of the transport-related factors on the health status of calves in the current experiment.

### Behavior

Behavior of calves is influenced by transport (11, 32). In the current study, calves spent more time lying than standing during transport, which was similar to other studies. Eicher and Morrow (33) showed that calves had a preference for lying (70% of the trip duration). Knowles et al. (22) reported that young calves (<1 month old) spent ~80% of their time lying down during 24 h transport duration. Overall, young calves prefer to lie more during transport compared to adult cattle (34), thus space requirements should account for these preferences. Calves not only showed more lying behavior during transport, but also directly post-transport and up to 24 h post-transport calves spent most of their time lying than standing. This suggests that transported calves might have experienced stress coupled with fatigue after the journey (32). Standing behavior almost doubled a week post-transport, suggesting that calves were beginning to recover from the journey. Calves mainly showed signs of discomfort the day and the week after transport, suggesting that transport caused a disturbance in their homeostasis and calves were able to cope with this challenge toward the end of this period. On the day post-transport, the highest prevalences of discomfort behavior were shown by calves transported in the conditioned truck and by calves transported for 18 h. Apparently, and intuitively logically, long-term transport (18 h) was more challenging to calves than short-term transport (6 h). The fact that calves transported in the conditioned truck exhibited more discomfort behavior in comparison with animals transported in the open truck warrants specific attention. This finding would suggest that transporting calves in a conditioned truck may be favorable for some health characteristics (such as naval inflammation, see above), but unfavorable in terms of behavioral signs of discomfort on the day post-transport. Again, this underlines the need for further research on conditioned transport, and its effect on calf health and behavior. Beyond the first week post-transport, discomfort behavior declined and the gradual increase in comfort behavior might be an indication that calves were adapting to the new environment. Playing behavior significantly increased until week 9; beyond this age the prevalence of this behavior remained relatively low. These changes might be age-related, but may also have been affected by the reduction in space availability in the pen (35). Transport-related factors did not significantly affect veal calf behavior from week 5 until 13; thus, similar to health, the various transport-related factors examined in the present study seemed to exert significant effects on behavior in the short term only.

### Slaughter Characteristics

In the current study, transport-related factors had no significant effect on either carcass weight, meat color, or conformation class. Notably, carcass weight was negatively related to the number of individual medical treatments. These results are in line with Pardon et al. (36) who demonstrated that antimicrobial drug use (ADU) was negatively associated with hot carcass weight of veal calves. Every increase in ADU by 1% was associated with 1.5 kg loss in hot carcass weight. Pardon et al. (36) also showed that carcass weight decreased severely with an increasing number of episodes of bovine respiratory disease and diarrhea. Moreover, Pardon et al. (36) showed that the odds for undesirable red meat color were lower with an increase in ADU (OR = 0.86 per percentage increase in ADU; 0.95-CI: 0.76–0.98; *P* < 0.05). This was in contrast with the results of the current experiment that revealed a tendency to darker meat color in calves treated >2 times with medicines. It can be hypothesized that calves which received more than two medical treatments were the ones that were more sick and lagging in condition.

## Conclusive Remarks

The current study shows that pre-transport diet and type of vehicle affected health and behavior of veal calves in the short term, but had no effects in the long run, including on slaughter characteristics. Perhaps transport-related effects were masked due to multiple use of medical treatments in the first weeks after arrival at the veal farm. Additionally, it might be assumed that the collective effects of the transition from the dairy farm to the veal farm, and of the husbandry conditions during the subsequent rearing period, on the adaptive capacity of calves were so large that the effects of individual transport-related factors were overruled. Despite the lack of treatment effects, the high prevalence of health problems merits more research on strategies to improve health of calves at the veal farm. Further studies are needed on ways to increase the resilience of veal calves during the transition from the dairy farm to the veal farm. These studies should also address transport-related factors in combination with (innovative) husbandry strategies both at the dairy farm and at the veal farm. Correspondingly, there is a need to define and record the (required and appropriate) environmental and climatic conditions and factors during conditioned transport of young calves, and to further study their relationship with calf health and welfare.

## Data Availability Statement

In principle, the raw data supporting the conclusions of this article are confidential. However, upon request to the corresponding author, data sharing will be considered after consultation with the stakeholders.

## Ethics Statement

The animal study was reviewed and approved by Central Committee on Animal Experiments (the Hague, the Netherlands; Approval Number 2017.D-0029).

## Author Contributions

FM wrote the manuscript and performed the statistical analyses. HB, BK, BE, MW-F, and KR contributed to manuscript revision, read, and approved the submitted version. All authors contributed to the conception and design of the study.

## Conflict of Interest

The authors declare that the research was conducted in the absence of any commercial or financial relationships that could be construed as a potential conflict of interest.

## References

[1] Hordijk J Veldman K Dierikx C van Essen-Zandbergen A Wagenaar JA Mevius D Prevalence and characteristics of quinolone resistance in *Escherichia coli* in veal calves. Vet Microbiol. (2012) 156:136–42. 10.1016/j.vetmic.2011.10.006 22041448

[2] Mormede P Soissons J Bluthe RM Raoult J Legarff G Levieux D. Effect of transportation on blood serum composition, disease incidence, and production traits in young calves. Influence of the journey duration. Ann Rech Vet. (1982) 13:369–84. 7185323

[3] Renaud D Kelton D LeBlanc S Haley D Duffield T. Calf management risk factors on dairy farms associated with male calf mortality on veal farms. J Dairy Sci. (2018) 101:1785–94. 10.3168/jds.2017-13578 29248230

[4] Autio T Pohjanvirta T Holopainen R Rikula U Pentikainen J Huovilainen A. Etiology of respiratory disease in non-vaccinated, non-medicated calves in rearing herds. Vet Microbiol. (2007) 119:256–65. 10.1016/j.vetmic.2006.10.001 17084565 PMC7130506

[5] Brscic M Leruste H Heutinck LF Bokkers EA Wolthuis-Fillerup M Stockhofe N. Prevalence of respiratory disorders in veal calves and potential risk factors. J Dairy Sci. (2012) 95:2753–64. 10.3168/jds.2011-4699 22541506

[6] Pempek J Trearchis D Masterson M Habing G Proudfoot K. Veal calf health on the day of arrival at growers in Ohio. J Anim Sci. (2017) 95:3863–72. 10.2527/jas.2017.1642 28992033

[7] Marcato F van den Brand H Kemp B van Reenen K. Evaluating potential biomarkers of health and performance in veal calves. Front Vet Sci. (2018) 5:133. 10.3389/fvets.2018.00133 29977895 PMC6021515

[8] Renaud D Duffield T LeBlanc S Ferguson S Haley D Kelton D Risk factors associated with mortality at a milk-fed veal calf facility: a prospective cohort study. J Dairy Sci. (2018) 101:2659–68. 10.3168/jds.2017-13581 29290439

[9] González L Schwartzkopf-Genswein K Bryan M Silasi R Brown F. Relationships between transport conditions and welfare outcomes during commercial long haul transport of cattle in North America. J Anim Sci. (2012) 90:3640–51. 10.2527/jas.2011-4796 22665659

[10] Sanderson MW Dargatz DA Wagner BA. Risk factors for initial respiratory disease in United States' feedlots based on producer-collected daily morbidity counts. Can Vet J. (2008) 49:373 18481546 PMC2275341

[11] Jongman EC Butler KL. The effect of age, stocking density and flooring during transport on welfare of young dairy calves in Australia. Animals. (2014) 4:184–99. 10.3390/ani4020184 26480036 PMC4494384

[12] Marcato F van den Brand H Kemp B Engel B Wolthuis-Fillerup M van Reenen K. Effects of pretransport diet, transport duration, and type of vehicle on physiological status of young veal calves. J Dairy Sci. (2020) 103:3505–20. 10.3168/jds.2019-17445 32037174

[13] SBK (2018). Available online at: https://www.kalversector.nl/wp-content/uploads/2018/03/SBK-KVK-BIJL-700-02-20180401-Protocol-Gezonde-Kalveren.pdf (in Dutch, accessed December 9, 2019).

[14] Webb LE Bokkers EAM Heutinck LFM Engel B Buist WG Rodenburg TB. Effects of roughage source, amount, and particle size on behavior and gastrointestinal health of veal calves. J Dairy Sci. (2013) 96:7765–76. 10.3168/jds.2012-6135 24094537

[15] Engel B Buist W van Reenen CG Housing of Calves in Experimental Facilities in Relation to Accuracy of Comparison of Feed Rations in Terms of Confidence Interval Length and Power of a Significance Test. Confidential report, commissioned by Denkavit Nederland BV (2016).

[16] Martin P Bateson PPG Bateson P Measuring Behaviour: An Introductory Guide. Cambridge: Cambridge University Press (1993).

[17] Webb LE Bokkers EA Engel B Gerrits WJ Berends H van Reenen CG Behaviour and welfare of veal calves fed different amounts of solid feed supplemented to a milk replacer ration adjusted for similar growth. Appl Anim Behav Sci. (2012) 136:108–16. 10.1016/j.applanim.2011.12.004

[18] European Community Commission Regulation (EC) no 1215/2003 of 7 July2003 amending Regulation (EEC) no 344/91 laying down detailed rules for applying Council Regulation (EEC) no 1186/90 to extend the scope of the community scale for the classification of carcasses of adult bovine animals. OJEC. (2003) 169/L:32–6. Available online at: https://eur-lex.europa.eu/legal-content/EN/TXT/PDF/?uri=CELEX:32003R1215&from=DE

[19] Breslow NE Clayton DG Approximate inference in generalized linear mixed models. J Am Stat Assoc. (1993) 88:9–25. 10.1080/01621459.1993.10594284

[20] Kenward MG Roger JH. Small sample inference for fixed effects from restricted maximum likelihood. Biometrics. (1997) 53:983–97. 10.2307/2533558 9333350

[21] Wilson L Smith J Smith D Swanson D Drake T Wolfgang D. Characteristics of veal calves upon arrival, at 28 and 84 days, and at end of the production cycle1. J Dairy Sci. (2000) 83:843–54. 10.3168/jds.S0022-0302(00)74948-4 10791802

[22] Knowles T Warriss P Brown S Edwards J Watkins P Phillips A Effects on calves less than one month old of feeding or not feeding them during road transport of up to 24 hours. Vet Rec. (1997) 140:116–24. 10.1136/vr.140.5.116 9042695

[23] Renaud D Duffield T LeBlanc S Haley D Kelton D. Clinical and metabolic indicators associated with early mortality at a milk-fed veal facility: a prospective case-control study. J Dairy Sci. (2018) 101:2669–78. 10.3168/jds.2017-14042 29290429

[24] Schaefer AL Jones SDM Stanley RW. The use of electrolyte solutions for the reducing transport stress. J Anim Sci. (1997) 75:258–65. 10.2527/1997.751258x 9027574

[25] Booth A Naylor J Correction of metabolic acidosis in diarrheal calves by oral administration of electrolyte solutions with or without bicarbonate. J Am Vet Med A. (1987) 191:62–8. 3038807

[26] Brown E VandeHaar M Daniels K Liesman J Chapin L Keisler D. Effect of increasing energy and protein intake on body growth and carcass composition of heifer calves. J Dairy Sci. (2005) 88:585–94. 10.3168/jds.S0022-0302(05)72722-3 15653525

[27] Quigley J Wolfe T Elsasser T. Effects of additional milk replacer feeding on calf health, growth, and selected blood metabolites in calves. J Dairy Sci. (2006) 89:207–16. 10.3168/jds.S0022-0302(06)72085-9 16357284 PMC7164769

[28] Mee JF. Newborn dairy calf management. Vet Clin N Am Food A. (2008) 24:1–17. 10.1016/j.cvfa.2007.10.002 18299029

[29] Pardon B De Bleecker K Dewulf J Callens J Boyen F Catry B. Prevalence of respiratory pathogens in diseased, non-vaccinated, routinely medicated veal calves. Vet Rec. (2011) 169:278. 10.1136/vr.d4406 21831999

[30] Pardon B Alliet J Boone R Roelandt S Valgaeren B Deprez P. Prediction of respiratory disease and diarrhea in veal calves based on immunoglobulin levels and the serostatus for respiratory pathogens measured at arrival. Prev Vet Med. (2015) 120:169–76. 10.1016/j.prevetmed.2015.04.009 25937168 PMC7114331

[31] Pardon B De Bleecker K Hostens M Callens J Dewulf J Deprez P. Longitudinal study on morbidity and mortality in white veal calves in Belgium. BMC Vet Res. (2012) 8:26. 10.1186/1746-6148-8-26 22414223 PMC3366893

[32] Grigor PN Cockram MS Steele WB Le Sueur CJ Forsyth RE Guthrie JA Effects of space allowance during transport and duration of mid-journey lairage period on the physiological, behavioural and immunological responses of young calves during and after transport. Anim Sci. (2001) 73:341–60. 10.1017/S135772980005832X

[33] Eicher S Morrow J Behavior following subcutaneous electrolyte treatment in transported calves. In: Ramos A Pinheiro Machado LC Hötzel MJ editors. Proceedings of the 34th International Congress of International Society of Applied Ethology (ISAE). Florianopolis: UFSC, Laboratory of Applied Ethology (2000). p. 76.

[34] Eicher S Transportation of cattle in the dairy industry: current research and future directions. J Dairy Sci. (2001) 84:E19–23. 10.3168/jds.S0022-0302(01)70192-0

[35] Rushen J de Passillé AM Locomotor play of veal calves in an arena: are effects of feed level and spatial restriction mediated by responses to novelty? Appl Anim Behav Sci. (2014) 155:34–41. 10.1016/j.applanim.2014.03.009

[36] Pardon B Hostens M Duchateau L Dewulf J De Bleecker K Deprez P. Impact of respiratory disease, diarrhea, otitis and arthritis on mortality and carcass traits in white veal calves. BMC Vet Res. (2013) 9:79. 10.1186/1746-6148-9-79 23587206 PMC3639957

