# Supplementary material for: Transport of Young Veal Calves: Effects of Pre-transport Diet, Transport Duration and Type of Vehicle on Health, Behavior, Use of Medicines, and Slaughter Characteristics
Source: Front Vet Sci. 2020 Dec 18;7:576469. doi: 10.3389/fvets.2020.576469 (PMC7775590; doi:10.3389/fvets.2020.576469)
Supplement: Supplementary file 1 [file Table_1.docx]

**Appendix 1**

Mean and range (between brackets) of actual temperature (T) and relative humidity (RH) inside the conditioned and open trucks during short (6 hours) or long (18 hours) transport of young calves to the veal farm.

|  | Conditioned truck | | | | Open truck | | | |
| --- | --- | --- | --- | --- | --- | --- | --- | --- |
|  | Batch 1 | | Batch 2 | | Batch 1 | | Batch 2 | |
|  | T (°C) | RH (%) | T (°C) | RH (%) | T (°C) | RH (%) | T (°C) | RH (%) |
| 6 h | 9.2  (8.2 – 10.3) | 66.0  (61.0 – 75.1) | 13.0  (11.7 – 13.9) | 74.1  (65.0 – 81.0) | 7.4  (6.2 – 9.1) | 74.1  (66.3 – 84.2) | 11.5  (10.4 – 12.5) | 80.3  (67.6 – 88.8) |
| 18 h | 7.8  (4.5 – 11.2) | 68.2  (58.5 – 78.9) | 13.6  (11.2 – 16.3) | 77.9  (65.4 – 83.9) | 6.6  (3.9 – 9.6) | 75.8  (66.3 – 86.5) | 14.0  (10.8 – 16.6) | 77.3  (66.2 – 86.2) |
