# Supplementary material for: Transport of Young Veal Calves: Effects of Pre-transport Diet, Transport Duration and Type of Vehicle on Health, Behavior, Use of Medicines, and Slaughter Characteristics
Source: Front Vet Sci. 2020 Dec 18;7:576469. doi: 10.3389/fvets.2020.576469 (PMC7775590; doi:10.3389/fvets.2020.576469)
Supplement: Supplementary file 2 [file Table_2.docx]

**Appendix 2**

Health parameters of veal calves assessed at the collection center and at the veal farm (from day 1 until week 3 post-transport). Health parameters were assessed at individual level.

| **Health parameter** | **Score** | **Explanation** |
| --- | --- | --- |
| Navel inflammation | 0 | No signs of inflammation |
|  | 1 | Swollen, without discharge |
|  | 2 | Swollen, with discharge |
| Joint inflammation | 0 | No evidence of joint problems |
|  | 1 | Evidence of joint inflammation |
| Loose or liquid manure | 0 | No loose or liquid manure |
|  | 1 | Pasty manure |
|  | 2 | Watery manure |
| Pneumonia | 0 | No abnormal breathing, no cough |
|  | 1 | Abnormal breathing and cough |
| Eye discharge | 0 | No eye discharge |
|  | 1 | Slight watery discharge |
| Sunken eyes | 0 | Normal, bright eyes |
|  | 1 | Eyes markedly recessed into the orbits |
| Ears | 0 | Normal |
|  | 1 | Drooped-eared |
| Nasal discharge | 0 | No discharge |
|  | 1 | Watery discharge |
|  | 2 | Purulent discharge |
