# Supplementary material for: Transport of Young Veal Calves: Effects of Pre-transport Diet, Transport Duration and Type of Vehicle on Health, Behavior, Use of Medicines, and Slaughter Characteristics
Source: Front Vet Sci. 2020 Dec 18;7:576469. doi: 10.3389/fvets.2020.576469 (PMC7775590; doi:10.3389/fvets.2020.576469)
Supplement: Supplementary file 3 [file Table_3.docx]

**Appendix 3**

Health parameters of veal calves assessed at pen level from week 5 until week 27 post-transport.

| **Health parameters** | **Explanation** | **Method of assessment** |
| --- | --- | --- |
| Milk leftovers | Untouched rests of milk in the feeding trough | Yes / No |
| Roughage leftovers | Untouched rests of roughage in the feeding trough | Yes / No |
| Abnormal breathing | Fast breathing (> 40 breaths/min), excessive abdominal breathing | number of calves |
| Nose discharge | Presence of discharge from one or both nostrils | number of calves |
| Coughing | Audible expulsion of air through the mouth of calves | number of calves |
| Loose or liquid manure | Presence of loose or liquid manure in the pen | Yes / No |
| Thick manure | Thicker and higher consistency manure, often combined with undigested food | Yes / No |
| White manure | Sticky and higher consistency manure. The colour is white or grey | Yes / No |
| Bloated calves | Calves with overfilled/bloated belly (upper, lower, right, left and all around) | number of calves |
| Lame calves | Calves with a different load/ or do not stand on one or more legs | number of calves |
| Claw problems | Red and swollen skin around the claw, often combined with lameness | number of calves |
| Joint problems | Clear thickening of one or more joints caused by accumulation of fluids/ synovia. Often painful and combined with lameness | number of calves |
| Bursa problems | Clear thickening (disc or round shaped) of the joint. Usually not painful and calves are not lame | number of calves |
| Chewing wounds | Wounds (damaged tail/ ear or skin on the body) caused by other calves in the pen. | number of calves |
| Skin infection | Skin damage due to infection: presence of round, hairless spots and wrinkled skin | number of calves |
| Hard skin | Thickened skin (often wrinkled and hairless), especially on the withers | number of calves |
| Urine suckling | Calves that suckle urines from other calves as well as calves being suckled | number of calves |
| Condition 15-30 | Calves that are 15-30% behind condition (based on weight and size of calves) compared to the other calves in the herd | number of calves |
| Condition >30 | Calves that are >30% behind condition (based on weight and size of calves) compared to the other calves in the herd | number of calves |
| Wet fur | Calves with a wet fur all along the back line | number of calves |
| Dull fur | Dull fur with abnormal structure, gloss and length | number of calves |
| Sick calves | Calves (not scored earlier) that give a general sick impression, depressed calves/ not attentive | number of calves |
