# Supplementary material for: Transport of Young Veal Calves: Effects of Pre-transport Diet, Transport Duration and Type of Vehicle on Health, Behavior, Use of Medicines, and Slaughter Characteristics
Source: Front Vet Sci. 2020 Dec 18;7:576469. doi: 10.3389/fvets.2020.576469 (PMC7775590; doi:10.3389/fvets.2020.576469)
Supplement: Supplementary file 4 [file Table_4.docx]

**Appendix 4**

| **Behavior** | **Description** |
| --- | --- |
| *Posture* |  |
| Stand | Body elevated from floor and weight supports by legs. |
| Lie | Brisket in contact with the floor. |
| *Activity* |  |
| Tongue play | Tongue playing/rolling. Turning, rolling and unrolling of tongue extended outside or inside mouth. |
| Urine drink | Preputial sucking of pen mates ore drinking of urine flow. |
| Chew | Chewing/ruminating. Any repetitive movements of lower jaw in lateral plane. |
| Manipulate objects | Oral manipulation of the pen structure and bucket/trough. |
| Lick another calf | Tongue extending and shifted across any body part of pen mate or nibbling of pen mate. |
| Manipulate another calf | Sucking any body parts (ears, tail, face, body) of pen mate excluding prepuce). |
| Graze | Grazing pen mate hair situated on back. Mouth nibbles and pulls away from back, sometimes coming away with hair. |
| Self-groom | Tongue extending and shifted across any body part of self or nibbling of self. |
| Rub | Rub any part of body against substrate. |
| Leap/Jump/Buck/Turn | Forelegs lifted from ground, forepart of body elevated, with or without forward movement, kicking and turning (frolic behavior). |
| Mount | Mount other calf from any side. |
| Head-butt | Butting of substrate or another calf. |
| Head-shake | Head shaken or rotated. |
| Run | Rapid movement forward with all four legs leaving the floor at one point in time. |
| Sniff | Sniff at surroundings including calves. |
| Repetitive calling | Repetitive calling with apparent no reason. |
| Walking | Walk around the pen. |
| Eat straw | Eating straw in the pen. |
| Drink water | Drink water from the bucket (from week 3 at the veal farm). |
| Other | Any other activity not mentioned above. |

Ethogram used to assess behavior of veal calves at the collection center and at the veal farm.
